# Um Caleidoscópio Bioquímico Chamado Troponina

**DOI:** 10.36660/abc.20230031

**Published:** 2023-03-01

**Authors:** Fabrício Braga

**Affiliations:** 1 Laboratório de Performance Humana Rio de Janeiro RJ Brasil Laboratório de Performance Humana, Rio de Janeiro, RJ – Brasil; 2 Casa de Saúde São José Rio de Janeiro RJ Brasil Casa de Saúde São José, Rio de Janeiro, RJ – Brasil; 3 Confederação Brasileira de Triathlon Vila Velha ES Brasil Confederação Brasileira de Triathlon, Vila Velha, ES – Brasil

**Keywords:** Doenças Cardiovasculares, Síndrome Coronariana Aguda, COVID-19, Pandemia, Prognóstico/tendências, Biomarcadores, Troponina/metabolismo

*“Não existe essa coisa de novas ideias. Nos simplesmente tomamos um monte de velhas ideias e as colocamos em um tipo de caleidoscópio mental”*

A frase acima atribuída ao colossal escritor americano, Mark Twain (1835-1910) me traz uma provocação um tanto quanto incomodativa. As coisas sempre estiverem aqui. Tudo que é novo sempre esteve aqui e passa a “existir” até alguém olhe o que é antigo de um jeito diferente. Por exemplo, energia elétrica sempre esteve aqui, mas para evoluir de misticismo a utilidade doméstica muitos tiveram que ver esse fenômeno de forma diferente.

Dentre as etapas da propedêutica médica o prognóstico, essa propriedade de um juízo antecipado da evolução de uma condição clínica, é, a meu ver, a mais desafiadora. Avanços tecnológicos em diversas áreas permitiram um crescimento na habilidade diagnóstica do médico. Porém, junto como ela, veio a angústia do que fazer, que frequentemente se apodera do pensamento do médico mesmo em seus momentos de descanso. Medico ou não medico? Opero ou não opero? Coloco no CTI ou no quarto? Dou boas notícias a família ou preparo-os para um destino ruim de seu ente querido? Portanto, instrumentos que tragam a valor presente a evolução clínica são de extrema valia na medicina de precisão.

Os biomarcadores cardíacos sorológicos promoveram uma verdadeira revolução, não só no diagnóstico quanto no prognóstico das síndromes coronarianas agudas (SCA).^1^ Introduzida na prática clínica no final dos anos 50, a transaminase oxalacética (TGO) rapidamente foi incorporada na definição de infarto da Organização Mundial de Saúde.^2^ Ao longo dos anos 70, lactato desidrogenase (LDH) e creatinofosfoquinase (CPK) passaram também a ser utilizados como biomarcadores. A todos faltavam uma propriedade importante a testes diagnósticos: especificidade, uma vez que estando presentes no músculo esqueléticos, também se elevam nos danos musculares. No início da década de 80, a fração MB da CPK (CK-MB), mais prevalente no músculo cardíaco que no esquelético, promoveu modestos avanços na especificidade.^3^

Em meados da década de 90, ainda como residente de cardiologia do Hospital Universitário Pedro Ernesto (UERJ), vivi com entusiasmo a chegada de um novo marcador diagnóstico para as SCA: a Troponina. Rápida, sensível e bem mais específico, foi uma verdadeira revolução na abordagem diagnóstica e prognóstica das doenças cardiovascular. Passamos a poder ver aquele paciente com dor torácica e ECG normal na sala de emergência de um outro jeito, embora eles sempre estivessem lá.

Mas o século XXI reservava uma importância ainda maior a Troponina. Seu papel na avaliação prognóstica ampliou-se a várias situações. Da doença pulmonar obstrutiva crônica^4^ a insuficiência renal.^5^ De pacientes críticos^6^ a corredores de ultramaratona.^7^ Pacientes clinicamente semelhantes, passaram a ser vistos com detentores de diferentes histórias naturais de suas doenças pelo “olhar” da Troponina.

Desde a Segunda Guerra mundial, a pandemia de COVID-19 foi o maior desafio imposto a humanidade. Do ponto de vista sanitário, talvez o maior desafio na idade contemporânea. Com um número exponencialmente crescente de casos que lotaram unidades hospitalares, estratificar risco passou a ser relevante como nunca, visto que os escassos leitos de terapia intensiva passaram a ser preciosos.

Já tendo mostrado seu potencial prognóstico na epidemia de Influenza A (H1N1) em 2009,^8^ rapidamente começaram a surgir publicações evidenciando que, mais uma vez, a Troponina poderia estratificar o risco nos pacientes hospitalizados com COVID-19.^9,10^

Neste número dos Arquivos Brasileiros de Cardiologia, Barbosa et al.,^11^ nos trazem mais uma evidência do valor prognóstico da Troponina em pacientes com COVID-19. Numa coorte derivada do Brazilian COVID-19 Registry, contendo 2.925 indivíduos admitidos em 31 hospitais, de 17 cidades brasileiras, elevações de Troponina (troponina I ou T> percentil 99%) nas primeiras 24 horas após admissão hospitalar mais que dobraram o risco de morrer (RR 2,03, IC 95% 1,60-2,58) e aumentaram em 87% a necessidade de ventilação mecânica. De certo que pacientes mais velhos e com mais comorbidades apresentavam uma incidência maior de elevação de troponina. Todavia, após elegante ajuste estatístico, o potencial preditor da Troponina se manteve.

Diversos mecanismos são propostos para a elevação de Troponina na COVID-19: interação do SARS-CoV2 com receptor da enzima conversora da angiotensina, presente no miócito cardíaco, resposta inflamatória sistêmica, injúria imunomediada e etc. Mas se embora, o que faz suas concentrações séricas subirem ainda não esteja claro, não há mais dúvida de que uma Troponina elevada em pacientes com COVID-19 determina um prognóstico pior. Novamente, a presença de um valor elevado de Troponina nas primeiras 24 horas nos permitia “enxergá-lo” de uma forma diferente.

Otimista, creio que tenhamos evoluído como sociedade após esses 3 anos de pandemia, e o que porventura tenhamos aprendido nos permita mitigar as chances de algo semelhante acontecer novamente. Mas é certo que novas doenças infecciosas irão aparecer. Novas epidemias, quiçá pandemias desafiarão os médicos do futuro, e estes precisarão de novas ideias, feitas de ideias velhas olhadas pelo caleidoscópio de uma mente criativa. Que não se esqueçam de dosar a troponina.
