# Supplementary material for: Um Caleidoscópio Bioquímico Chamado Troponina
Source: Arq Bras Cardiol. 2023 Mar 1;120(2):e20230031. [Article in Portuguese] doi: 10.36660/abc.20230031 (PMC12080596; doi:10.36660/abc.20230031)
Supplement: Translation

# Translation: A Biochemical Kaleidoscope Called Troponin

**Authors:** Fabrício Braga

**Affiliations:** 1 Brazil Laboratório de Performance Humana, Rio de Janeiro, RJ – Brazil; 2 Brazil Casa de Saúde São José, Rio de Janeiro, RJ – Brazil; 3 Brazil Confederação Brasileira de Triathlon, Vila Velha, ES – Brazil

**Keywords:** Cardiovascular Diseases, Acute Coronary Syndrome, COVID-19, Pandemic, Prognosis/trends, Biomarkers, Troponin/metabolism

*“There is no such thing as new ideas. We just take a bunch of old ideas and put them into a kind of mental kaleidoscope.”*

The phrase above attributed to the colossal American writer Mark Twain (1835-1910) brings me a somewhat disturbing provocation. Things are always here. Everything new has always been here and starts to “exist” until someone looks at what is old differently. For example, electrical energy has always been here, but to evolve from mysticism to domestic utility, many had to see this phenomenon differently.

Among the stages of medical propaedeutics, prognosis, this property of an anticipated judgment of the evolution of a clinical condition, is, in my view, the most challenging. Technological advances in several areas have allowed the physician’s diagnostic ability to grow. However, along with it came the anguish of what to do, which often takes over the doctor’s thoughts even in his moments of rest. To prescribe or not to prescribe? To operate or not operate? Do I put it in the ICU or the room? Do I give the family good news or prepare them for a bad fate for their loved one? Therefore, instruments that bring clinical evolution to present value are extremely valuable in precision medicine.

Serological cardiac biomarkers promoted a real revolution, not only in the diagnosis but also in the prognosis of acute coronary syndromes (ACS).^1^ Introduced into clinical practice in the late 1950s, oxaloacetic transaminase (GOT) was quickly incorporated into the World Health Organization’s definition of infarction.^2^ Throughout the 1970s, lactate dehydrogenase (LDH) and creatine phosphokinase (CPK) also began to be used as biomarkers. All tests lacked an important property: specificity; since they are present in skeletal muscle, they also increase muscle damage. In the early 1980s, the MB fraction of CPK (CK-MB), more prevalent in cardiac muscle than skeletal muscle, promoted modest advances in specificity.^3^

In the mid-1990s, still as a cardiology resident at the Hospital Universitário Pedro Ernesto (UERJ), I experienced the arrival of a new diagnostic marker for ACS with enthusiasm: Troponin. Fast, sensitive, and much more specific, it was a true revolution in cardiovascular disease diagnostic and prognostic approach. We could see patients with chest pain and normal ECG in the emergency room in a different way, even though they were always there.

Nevertheless, the 21st century reserved even greater importance for Troponin. Its role in prognostic evaluation has extended to several situations. From chronic obstructive pulmonary disease^4^ to renal failure.^5^ From critical patients^6^ to ultramarathon runners.^7^ Clinically similar patients began to be seen as holders of different natural histories of their diseases by the “look” of Troponin.

Since World War II, the COVID-19 pandemic has been humanity’s greatest challenge. From a sanitary point of view, perhaps the greatest challenge in the contemporary age. With an exponentially growing number of cases filling hospital units, stratifying risk has become more relevant than ever as scarce intensive care beds have become precious.

Having already shown its prognostic potential in the Influenza A (H1N1) epidemic in 2009,^8^ publications quickly began to appear, showing that, once again, Troponin could stratify risk in patients hospitalized with COVID-19.^9,10^

In this number of the *Arquivos Brasileiros de Cardiologia*, Barbosa et al.^11^ bring us more evidence of the prognostic value of Troponin in patients with COVID-19. In a cohort derived from the Brazilian COVID-19 Registry, containing 2,925 individuals admitted to 31 hospitals from 17 Brazilian cities, elevations of Troponin (Troponin I or T > 99% percentile) in the first 24 hours after hospital admission more than doubled the risk of dying (RR 2.03, 95% CI 1.60-2.58) and increased the need for mechanical ventilation by 87%. Certainly, older patients with more comorbidities had a higher incidence of troponin elevation. However, after elegant statistical adjustment, Troponin’s predictive potential was maintained.

Several mechanisms are proposed for the elevation of Troponin in COVID-19: interaction of SARS-CoV2 with angiotensin-converting enzyme receptor, present in cardiac myocytes, systemic inflammatory response, immune-mediated injury, etc. However, although what causes their serum concentrations to rise is still unclear, there is no doubt that an elevated Troponin in patients with COVID-19 determines a worse prognosis. Again, a high Troponin value in the first 24 hours allowed us to “see” it differently.

Optimistic, I believe that we have evolved as a society after these 3 years of the pandemic and that perhaps we have learned to allow us to mitigate the chances of something similar happening again. Nevertheless, new infectious diseases will certainly appear. New epidemics, perhaps pandemics, will challenge future doctors, and they will need new ideas made from old ideas seen through the kaleidoscope of a creative mind. Do not forget to measure the Troponin.
